# Supplementary material for: Medics as a Positive Deviant in Influenza Vaccination: The Role of Vaccine Beliefs, Self-Efficacy and Contextual Variables
Source: Vaccines (Basel). 2022 May 5;10(5):723. doi: 10.3390/vaccines10050723 (PMC9148145; doi:10.3390/vaccines10050723)
Supplement: Supplementary file 1 [file vaccines-10-00723-s001.zip › vaccines-1653651-supplementary.pdf]

VACCINATION MULTI-MODULE QUESTIONNAIRE (VMMQ)

D. Włodarczyk, U. Ziętalewicz

GENERAL VACCINATION BELIEFS SCALE

Please indicate the extent to which you agree or disagree with the statements concerning vaccination presented below. Please rate every statement using a 5 point scale ranging from 1- strongly disagree; 2- rather disagree; 3- hard to tell; 4- rather agree; 5- strongly agree

1. It is safer to get vaccinated against influenza than to get it.
2. The side effects of influenza vaccination are more harmful than its benefits.
3. Influenza vaccination is a scientifically proven and well-researched way to reduce the spread of a preventable disease.
4. Vaccination against influenza is unnecessary because influenza is not a common and / or serious disease.
5. By vaccinating against the flu, you protect the health and life of people who, for health reasons, cannot be vaccinated.
6. Promotion of influenza vaccination is in the interest of narrow lobbing groups (e.g. pharmaceutical companies, corrupt scientists) that pursue their interests at the expense of human health.

RISK PERCEPTION SCALE

Please rate every statement using a 5 point scale ranging from 1- definitely lower risk than others; 2- rather lower; 3- same; 4- rather higher; 5- definitely higher risk than others

Compared to others of the same sex and age as you, how would you estimate the likelihood that in your current situation:

1. … you will catch the flu.
2. … If you catch the flu, you will have a severe course of it (eg. high fever, weakness, muscle aches, long duration of symptoms).
3. … If you catch the flu, you will develop severe complications such as pneumonia or a heart attack and / or hospital stay.
4. … you will die if you catch the flu.

OUTCOME EXPECTATIONS – PERCEIVED VACCINATION GAINS SCALE

Please indicate the extent to which you agree or disagree with the following statements in favour of influenza vaccination.

Please rate every statement using a 5 point scale ranging from 1- strongly disagree; 2- rather disagree; 3- hard to tell; 4- rather agree; 5- strongly agree

1. Influenza vaccination reduces my risk of getting the virus and/or getting the flu.
2. My risk of influenza complications, e.g. pneumonia, heart disease, is reduced.
3. The risk that people around me become infected (e.g. with whom I live and / or work) is reduced.
4. I'll save on treatment.
5. I feel that I care of my health.
6. I am setting a good example for others.
7. My protection is increasing during the coronavirus pandemic.
8. I protect those who cannot be vaccinated due to contraindications.

OUTCOME EXPECTATIONS - PERCEIVED VACCINATION LOSSES SCALE

Please indicate the extent to which you agree or disagree with the following statements against influenza vaccination.

Please rate every statement using a 5 point scale ranging from 1- strongly disagree; 2- rather disagree; 3- hard to tell; 4- rather agree; 5- strongly agree

1. My relatives would criticize me or be dissatisfied with me.
2. I would be afraid of negative side effects for my health.
3. The influenza vaccine does not give me enough confidence that I will not catch the flu.
4. Flu vaccination formalities would take my time.
5. I would have to struggle with bad organization of the health care system.
6. I would have to repeat this vaccination every year.
7. I would have additional financial expenses.
8. I would feel like a "guinea pig".
9. My risk of infection increases or my prognosis worsens if I am infected with coronavirus.

VACCINATION FACILITATORS' RELEVANCE SCALE

Please rate every statement using a 5 point scale ranging from 1- definitely irrelevant to me; 2- rather irrelevant; 3- hard to tell; 4- rather irrelevant; 5- definitely relevant to me

Regardless of whether you vaccinate or not, does or could this factor favour your decision to vaccinate?

1. Publicly available information to remind you when and how to vaccinate.
2. An individual invitation / reminder (letter, e-mail, text message or other) about the dates and ways of getting vaccinated against influenza prepared by your health clinic or family doctor / general practitioner.
3. The clinic of your choice provides visible information whether and to what extent its employees are vaccinated against influenza.
4. Information that your GP and / or his relatives are regularly vaccinated against flu.
5. Conversation about influenza vaccination initiated by your GP /specialist.
6. Organization of influenza vaccination as a regular activity of an occupational medicine physician.
7. Employer incentives to vaccinate against influenza or promoting vaccinations in the workplace (e.g. vaccinations during working hours, reimbursement of vaccination costs).
8. Free of charge, state compulsory flu vaccination.
9. The threat of the coronavirus epidemic.

VACCINATION SELF-EFFICACY SCALE

Please rate every statement using a 5 point scale ranging from 1- definitely not sure; 2- rather not sure; 3- hard to tell; 4- rather sure; 5- definitely sure

To what extent are you sure that you will vaccinate in the current season even if…

1. … you have to pay in full or in part for the influenza vaccination.
2. … friends or the media tell you that this flu vaccine is harmful or unnecessary, or that it is does not give a 100% guarantee.
3. … you will need to find out where and how to get the flu vaccine.
4. … you will be overwhelmed by the excess of other things and responsibilities.
5. … vaccination will have to be rescheduled, for example due to a cold.
6. … it will be necessary to wait or make further attempts to make an appointment due to the reduced availability of vaccines during the coronavirus pandemic.

VACCINATION INTENTION SCALE

I. Do you usually get vaccinated against the flu? (please include the last 5 years)

1. I have never had a flu vaccine.
2. I vaccinated in the past, but I haven’t had the flu vaccine for some time.
3. I get the flu vaccinations irregularly.
4. I didn’t vaccinate in the past, but I have had the flu vaccine for some time.
5. I always / usually get the flu vaccine.

II. Did you get vaccinated against flu last season (2019/2020)?

1. yes
2. no

III. Are you getting the flu vaccine this season (2020/2021)?

1. I have already vaccinated.
2. I have a flu vaccination scheduled / appointment.
3. I plan to get vaccinated, but it is currently impossible due to the lack / difficult access to flu vaccines.
4. I am not planning an influenza vaccination due to the lack of / difficult access to vaccines.
5. I am not planning an influenza vaccination due to contraindications.
6. I'm not going to get vaccinated.
